# Biomechanical Comparison of Three Modified Kessler Techniques for Flexor Tendon Repair: Implications in Surgical Practice and Early Active Mobilization

**DOI:** 10.3390/jcm13195766

**Published:** 2024-09-27

**Authors:** Marlies Schellnegger, Alvin C. Lin, Judith C. J. Holzer-Geissler, Annika Haenel, Felix Pirrung, Andrzej Hecker, Lars P. Kamolz, Niels Hammer, Werner Girsch

**Affiliations:** 1 Division of Macroscopy and Clinical Anatomy, Gottfried Schatz Research Center, Medical University of Graz, 8036 Graz, Austria; alvin.lin@pmu.ac.at (A.C.L.); niels.hammer@medunigraz.at (N.H.); 2 COREMED—Cooperative Centre for Regenerative Medicine, Joanneum Research Forschungsgesellschaft mbH, 8036 Graz, Austrialars.kamolz@medunigraz.at (L.P.K.); 3 Division of Plastic, Aesthetic and Reconstructive Surgery, Department of Surgery, Medical University of Graz, 8036 Graz, Austria; judith.geissler@medunigraz.at (J.C.J.H.-G.); werner.girsch@uniklinikum.kages.at (W.G.); 4 Institute of Anatomy and Cell Biology, Paracelsus Medical University, 5020 Salzburg, Austria; 5 Department of Orthopedic and Trauma Surgery, University of Leipzig, 04103 Leipzig, Germany; 6 Division of Biomechatronics, Fraunhofer Institute for Machine Tools and Forming Technology IWU, 01187 Dresden, Germany

**Keywords:** flexor tendon repair, biomechanical properties, early active mobilization, Kessler–Tsuge, Kirchmayr–Kessler

## Abstract

**Objective**: Managing flexor tendon injuries surgically remains challenging due to the ongoing debate over the most effective suture technique and materials. An optimal repair must be technically feasible while providing enough strength to allow for early active mobilization during the post-operative phase. This study aimed to assess the biomechanical properties of three modified Kessler repair techniques using two different suture materials: a conventional two-strand and a modified four-strand Kirchmayr–Kessler repair using 3-0 Prolene^®^ (2s-KK-P and 4s-KK-P respectively), and a four-strand Kessler–Tsuge repair using 4-0 FiberLoop^®^ (4s-KT-FL). **Methods**: Human flexor digitorum profundus (FDP) tendons were retrieved from Thiel-embalmed prosections. For each tendon, a full-thickness cross-sectional incision was created, and the ends were reattached using either a 2s-KK-P (n = 30), a 4s-KK-P (n = 30), or a 4s-KT-FL repair (n = 30). The repaired tendons were tested using either a quasi-static (n = 45) or cyclic testing protocol (n = 45). Maximum force (F_max_), 2 mm gap force (F_2mm_), and primary failure modes were recorded. **Results**: In both quasi-static and cyclic testing groups, tendons repaired using the 4s-KT-FL approach exhibited higher F_max_ and F_2mm_ values compared to the 2s-KK-P or 4s-KK-P repairs. F_max_ was significantly higher with a 4s-KK-P versus 2s-KK-P repair, but there was no significant difference in F_2mm_. Suture pull-out was the main failure mode for the 4s-KT-FL repair, while suture breakage was the primary failure mode in 2s- and 4s-KK-P repairs. **Conclusions**: FDP tendons repaired using the 4s-KT-FL approach demonstrated superior biomechanical performance compared to 2s- and 4s-KK-P repairs, suggesting that the 4s-KT-FL tendon repair could potentially reduce the risk of gapping or re-rupture during early active mobilization.

## 1. Introduction

Flexor tendon injuries are amongst the most commonly treated conditions in hand surgery [1]. Surgical repair remains challenging, partly due to a lack of consensus on the ideal suture technique and material. An optimal repair should minimize friction and provide sufficient tensile strength to withstand an early active mobilization protocol in the post-operative phase [2]. Early active mobilization has been shown to significantly enhance functionality following tendon repair, with improved tendon healing and decreased peritendinous adhesions. Additionally, the repair should be technically feasible, allowing the technique to be performed in a standardized manner with minimal variability [3,4]. However, standardizing flexor tendon repair is challenged by variations in tendon anatomy, injury complexity, and tissue quality.

Often, the choice of surgical technique is influenced by the type of suture material, and vice versa [5], adding further complexity to achieving uniform repair results. A major challenge following tendon repair is balancing early mobilization with the risk of gap formation between the repaired tendon stumps. Thus, the ideal repair would require minimal material whilst remaining resistant to gap formation during active post-operative mobilization.

With this approach, various tendon repair techniques have been developed and tested over the past few decades [6]. The Kirchmayr–Kessler technique, as modified by Zechner [6], enhances repair strength whilst maintaining simplicity and allows for modifications, such as the addition of core suture strands, to reduce gapping during early mobilization, making it widely adopted in clinical practice [4,7]. Previous biomechanical studies have shown that increasing the number of core suture strands improves tensile strength. Therefore, a double-throw Kessler technique, which involves two suture strands through the core, is recommended for a more reliable repair. Using looped material such as FiberLoop^®^ (Arthrex, Naples, FL, USA) further simplifies this approach, as it provides two strands with each needle passage through the tendon core [2,8]. Biomechanical studies have demonstrated that FiberWire^®^ offers superior biomechanical properties compared to commonly used suture materials such as Prolene^®^ (Ethicon Inc.; Johnson & Johnson, Somerville, NJ, USA) or Ethibond^®^ (Ethicon Inc.; Johnson & Johnson, Somerville, NJ, USA) [9,10].

This study aimed to evaluate the biomechanical performance of three common flexor tendon repair techniques in vitro, all based on the Kessler technique that use different suture materials: the Kirchmayr–Kessler technique with single-stranded Prolene^®^ 3-0, evaluated for both a two-strand (2s-KK-P) and four-strand (4s-KK-P) repair, and the Kessler–Tsuge technique with looped FiberLoop^®^ 4-0, resulting in a four-strand repair (4s-KT-FL). The suitability for early active mobilization was assessed by analyzing their load–deformation properties under quasi-static and cyclic testing conditions. It was hypothesized that the Kessler–Tsuge technique with FiberLoop^®^ would yield superior biomechanical properties compared to the two Kirchmayr–Kessler approaches using Prolene^®^.

## 2. Materials and Methods

### 2.1. Materials

A total of 90 flexor digitorum profundus (FDP) tendons were retrieved from 28 Thiel-embalmed bodies (15 females, 13 males, mean age at death: 73 ± 6.4 years). All embalmed bodies were provided by the Medical University of Graz. Only FDP tendons of the index and middle fingers were retrieved to assure homogenous tissue properties [6,11]. The laterality of the limbs was unknown. Bodies with upper extremity pathologies or other known history of musculoskeletal pathologies were excluded. The procedures were approved by the Ethics Committee of the Medical University of Graz (approval number: 33-189 ex 20/21).

### 2.2. Methods

#### 2.2.1. Study Design

FDP tendons were randomly assigned to one of the three suture repair techniques using a simple randomization process, ensuring unbiased allocation across all groups: the conventional 2-strand Kirchmayr–Kessler technique with Prolene^®^ 3-0 (2s-KK-P), the modified 4-strand Kirchmayr–Kessler technique with Prolene^®^ 3-0 (4s-KK-P), and the 4-strand Kessler–Tsuge technique with Fiberloop^®^ (4s-KT-FL). All tendon sutures were performed by two experienced surgeons. The tendon repairs were equally and randomly assigned to two testing scenarios, a quasi-static or a cyclic protocol. Thus, for each repair technique, 15 tendons were assigned to each of the two testing protocols, resulting in a total of 45 tendons tested under the quasi-static protocol and 45 tendons under the cyclic protocol. Both testing protocols were based on protocols used in previous studies [12,13]. Biomechanical testing was performed collaboratively by two investigators with experience in biomechanics. Working together throughout the testing process ensured consistency and minimized variability in the measurements.

#### 2.2.2. Suture Technique for Tendon Repair

To create a standardized tendon defect, a full-thickness transverse incision was made across the FDP tendon at the level corresponding to the middle part of zone 2 [14]. All tendons were length measured with a Vernier caliper (accuracy 0.05 mm). The core suture in the Kirchmayr–Kessler repair was set at 10 mm from the end of the tendon stump using 3-0 Prolene^®^. This technique consists of two separate single-loop sutures using the Kessler approach. The core stitches were placed in the middle plane of the tendon, and the transverse strand was placed approximately 7 mm from the margin. The 4-strand tendon repair with Prolene^®^ 3-0 was performed with a modified approach of the Kirchmayr–Kessler technique as described above but with the placement of an additional two stitches to attain 4 strands across the core. The depth and purchase length of the strands were essentially the same to ensure even tension and strength. However, there is typically a slight offset in the stitch placement to prevent the strands from passing through the exact same spot, which helps preserve tendon tissue integrity and avoid weakening the repair site.

**Figure 1:**
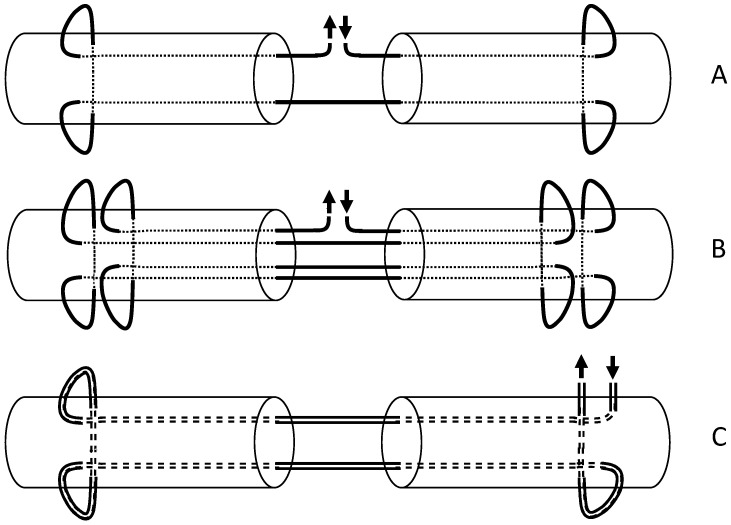
**Schematic representation of the tested tendon suture repairs.** (**A**) The conventional 2-stranded Kirchmayr–Kessler repair performed with Prolene^®^ 3-0, (**B**) the modified 4-stranded Kirchmayr–Kessler repair performed with Prolene^®^ 3-0, and (**C**) the Kessler–Tsuge repair performed with FiberLoop^®^ 4-0.

For the Kessler–Tsuge technique (2), a looped suture material (FiberLoop^®^) was used to create a 4-strand modification of the classic Kessler repair. The suture entered from the side of the tendon stump, passed through the tendon core, and exited on the same side. It was then passed back in the reverse direction, forming two parallel strands across the tendon repair. A schematic representation of all three repair approaches is given in Figure 1.

For the three suture techniques, an additional epitendinous running suture (5-0, Prolene^®^, Ethicon) was placed around the circumference of the repair, which is akin to clinical practice. This running suture consisted of approximately ten stitches at a distance of 1.5 mm or 3.0 mm across the repair and at depth of 1.0 mm. All sutures were performed with taper point and ½ circle needles.

#### 2.2.3. Biomechanical Testing

All tendon repairs were assessed on a uniaxial tensile testing device (Zwick Roell Z020 equipped with a 2500-N Xforce HP load cell, Ulm, Germany). Digital image correlation (Aramis, GOM, Brunswick, Germany), synchronized with the tensile testing device, was used to evaluate the opening of the suture gap under loading of the tendons. Further, the image correlation system was used to record the failure mode, which was described as suture pull-out, suture breakage, or slippage of the knot [13]. The system used offers a maximum resolution of 40,096 × 3000 pixels, providing high precision in capturing tendon deformation. The testing devices were calibrated according to the manufacturer’s standard protocols prior to testing to ensure the accuracy and consistency of the measurements. To avoid any slippage of the mounted tendons and to ensure a repeatable grip-to-grip distance, a self-developed clamping system was employed. The clamping system was 3D-printed, ensuring proper fixation without damaging the tissue. It was validated in a previous study through quasi-static and fatigue tests and scanning electron microscopy, confirming that no artifacts were introduced into the measurements [15]. With its help, the tendon repairs were placed in pneumatic clamps (ZwickRoell 8397) to ensure a constant clamping pressure.

For the quasi-static protocol, the samples were preconditioned for five cycles at a rate of 0.1 mm/s and 5 N to remove initial slack and align the tendon fibers, a process that ensures the reproducibility of the mechanical properties before testing [16,17]. Following the preconditioning, the samples were tested at a constant rate of 0.1 mm/s until the samples failed mechanically. The maximum force (F_max_) was recorded. A testing speed of 0.1 mm/s was selected to capture the true tensile properties under quasi-static conditions, minimizing the potential for strain rate-related artifacts.

For the cyclic protocol, the tendons underwent the same preconditioning as described for the quasi-static protocol. Subsequently, tendons were preloaded to 12 N and then pre-cycled between 3 and 20 N for 20 cycles at 0.2 Hz. The loading force was successively increased by 5 N increments, maintaining a constant force amplitude of 17 N. Each new loading magnitude was repeated for 20 cycles until a peak load of 80 N was reached, or the repair failed. If the suture was still intact following the last cycle, the tendons were pulled at a constant rate of 0.1 mm/s until rupture. In the cyclic protocol, F_max_, F_2mm_, the number of cycles at 2 mm gap formation (N_cycles_ at F_2mm_), and the number of completed cycles until rupture (N_cycles_ at F_max_) were recorded.

#### 2.2.4. Statistical Analyses

The data were analyzed with GraphPad Prism software (version 9.4.1; GraphPad Software, Inc., San Diego, CA, USA). Normal distribution was assessed using the Shapiro–Wilk test. A one-way ANOVA with the Tukey post hoc test was used to compare the metric variables. If the data did not follow a normal distribution, non-parametric alternatives were applied. For categorical variables, a Chi-Square test was used with Bonferroni correction. A Cramér’s V analysis was applied to assess the correlation between the different suture techniques and the failure mode. Considering the given four degrees of freedom, Ф of 0.05 indicates a weak, 0.15 a medium, and 0.25 a strong association [18]. All statistical tests were two-tailed with the alpha level set at 0.05. Values are reported as means ± standard deviations as well as minima and maxima.

## 3. Results

### 3.1. The Four-Strand Kessler–Tsuge FiberLoop^®^ Repair Yields a Higher Maximum Force than the Two- and Four-Strand Kirchmayr–Kessler Repair

Quasi-static testing of the two conventional Kirchmayr–Kessler suture techniques with Prolene^®^ 3-0 yielded different F_max_ values of 44.6 ± 5.3 N for the two-strand repair and 55.4 ± 6.5 N for the four-strand repair (*p* = 0.01). In contrast, the Kessler–Tsuge repair with FiberLoop^®^ 4-0 failed at an F_max_ of 68.4 ± 18.3 N, which was higher than the F_max_ for both two- and four-strand Kirchmayr–Kessler Prolene^®^ repairs (*p* ≤ 0.01 for both).

**Figure 2:**
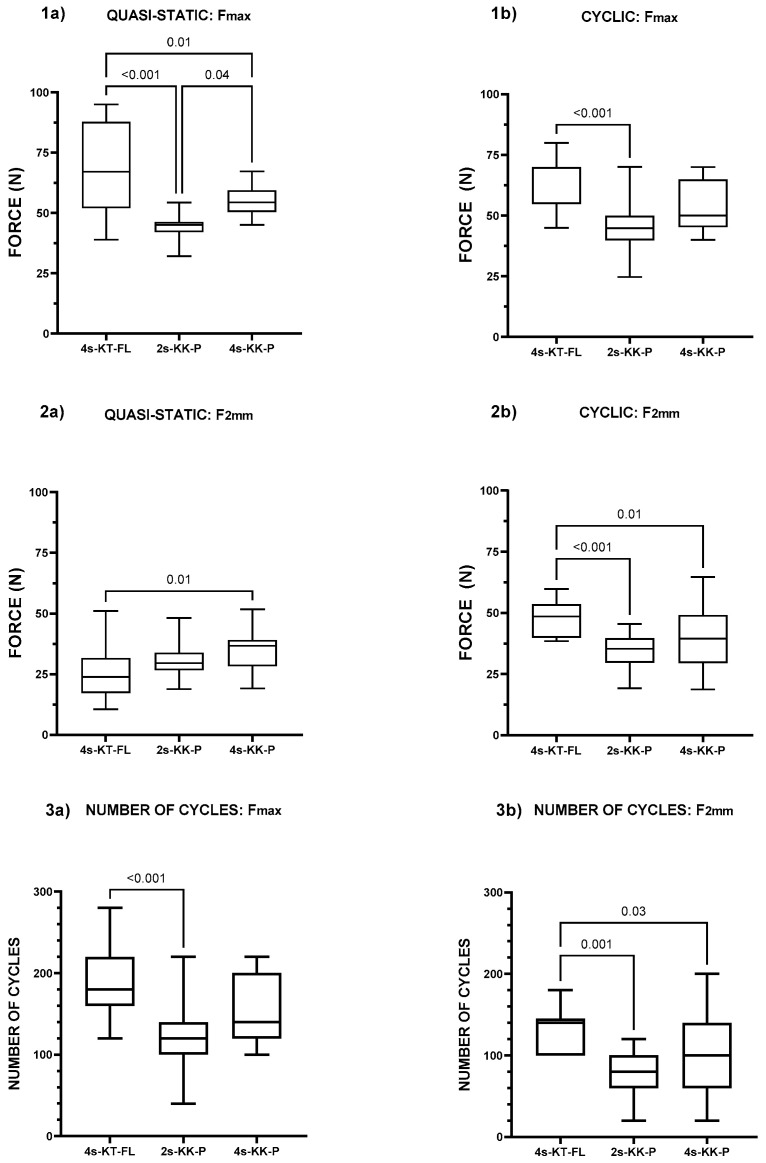
(**1**) The 4s-KT-FL repair yields a higher maximum force (F_max_). The F_max_ achieved using a (**a**) quasi-static protocol and (**b**) a cyclic protocol. (**2**) The 4s-KT-FL repair yields a higher force at 2 mm gap formation (F_2mm_) in cyclic testing. The F_2mm_ observed using a (**a**) quasi-static protocol and (**b**) a cyclic protocol. (**3**) The 4s-KT-FL repair yields a higher number of completed cycles (N_cycles_) using a cyclic protocol. The N_cycles_ achieved at (**a**) the maximum force or F_max_, and (**b**) the force at 2 mm gap formation (F_2mm_). 4s-KT-FL: 4-strand Kessler–Tsuge 4-0 FiberLoop^®^ repair; 2s-KK-P: 2-strandKirchmayr–Kessler 3-0 Prolene^®^ repair; 4s-KK-P: 4-strand Kirchmayr–Kessler 3-0 Prolene^®^ repair.

In cyclic testing, the F_max_ of the 4s-KT-FL repair averaged at 63.0 ± 10.4 N, which was higher when compared to the F_max_ of the 2s-KK-P repair of 46.1 ± 11.4 N (*p* < 0.001) and the 4s-KK-P repair at 54.2 ± 9.7 N (*p* = 0.07). However, amongst the two-strand and four-strand Kirchmayr–Kessler repairs, the F_max_ values were not statistically different (*p* = 0.1). The maximum force for each of the three repair groups and for both testing protocols is displayed in Figure 2.

### 3.2. The Four-Strand Kessler–Tsuge Fiberloop^®^ Repair Yields a Higher Force at 2 mm Gap Formation than the Two- and Four-Strand Kirchmayr–Kessler Repair in Cyclic Testing

For the 4s-KT-FL repair, F_2mm_ occurred at 48.2 ± 6.7 N in cyclic testing. For the 2s-KK-P repair, a 2 mm gap was observed at 35.5 ± 6.8 N, while the 4s-KK-P repair registered forces of 38.3 ± 12.1 N until a 2 mm gap formed. The minimum force to induce 2 mm gap formation was 38.5 N in the 4s-KT-FL repair group compared with 19.2 N and 18.8 N for the 2s-KK-P and 4s-KK-P repair groups, respectively. F_2mm_ was significantly higher with the 4s-KT-FL repair compared to the 2s-KK-P (*p* < 0.001) and 4s-KK-P (*p* < 0.01) repairs. A comparison of F_2mm_ between the two-strand and four-strand Kirchmayr–Kessler Prolene^®^ repairs yielded no significant difference (*p* = 0.67). The comparison of the applied forces until 2 mm gap formation occurred is shown in Figure 2.

### 3.3. The Four-Strand Kessler–Tsuge FiberLoop^®^ Repair Demonstrates a Higher Number of Completed Cycles and a Greater 2 mm Gap Force than the Two- and Four-Strand Kirchmayr–Kessler Repair

Tendons repaired by the Kessler–Tsuge technique completed on average 193.3 ± 47.6 cycles until F_max_ was reached, which was higher when compared to the two-strand Kirchmayr–Kessler repair, with 125.3 ± 45.0 completed cycles (*p* = 0.001). There was no significant difference in the number of completed cycles until F_max_ was reached between the four-strand Kessler–Tsuge and four-strand Kirchmayr–Kessler repairs (*p* = 0.07), with the latter enduring 157.3 ± 38.5 completed cycles. No difference was observed amongst the two Kirchmayr–Kessler techniques in completed cycles until F_max_ was reached (*p* = 0.13).

For the number of completed cycles until F_2mm_ was reached, the four-strand Kessler–Tsuge repair resulted in 131.4 ± 25.7 cycles, which was statistically significant when compared to either the two-strand or four-strand Kirchmayr–Kessler repairs, with 80.0 ± 30.2 cycles (*p* = 0.001) and 94.7 ± 48.1 cycles (*p* = 0.02), respectively. There was no significant difference in the number of completed cycles until F_2mm_ was reached between the two Kirchmayr–Kessler techniques (*p* = 0.51). In contrast to the number of cycles until F_max_, there was a significant difference observed in the number of completed cycles until F_2mm_ was reached between the four-strand Kessler–Tsuge FiberLoop^®^ and the four-strand Kirchmayr–Kessler repair (*p* = 0.002), with the latter registering fewer competed cycles.

The number of completed cycles until the maximum force and the force to achieve 2 mm gap formation were reached are shown in Figure 2.

### 3.4. Suture Pull-Out Was the Primary Failure Mode in the Four-Strand Kessler–Tsuge FiberLoop^®^ Repair and Suture Breakage in the Kirchmayr–Kessler Prolene^®^ Repairs

In the quasi-static testing, 87% (13/15) of the tendon repairs using the Kessler–Tsuge FiberLoop^®^ technique failed by suture pull-out, whilst 13% (2/15) of repairs failed due to the slippage of the knot. In contrast, 60% (9/15) of tendon repairs with the conventional two-strand Kirchmayr–Kessler technique failed by suture rupture or breakage, with 33% (5/15) of sutures failing by pull-out from the tendon tissue and 7% (1/15) failing by slippage of the knot. The primary failure mode of the double Kirchmayr–Kessler repair was suture breakage at 80% (12/15).

**Figure 3:**
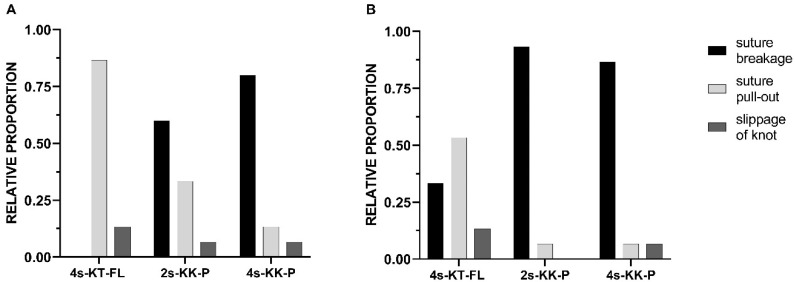
**The relative proportion of the primary failure mode** using a (**A**) quasi-static protocol and (**B**) a cyclic protocol. For the 4s-KT-FL repair, the primary failure mode was suture pull-out; for the 2s-KK-P and 4s-KK-P repairs, the primary failure mode was suture breakage. The 4s-KT-FL repair ((**A**) quasi-static protocol) had no suture breakage and the 2s-KK-P repair ((**B**) cyclic protocol) had no slippage of the knot; as a result, no value is reported. 4s-KT-FL: 4-strand Kessler–Tsuge 4-0 FiberLoop^®^ repair; 2s-KK-P: 2-strand Kirchmayr–Kessler Prolene^®^ repair; 4s-KK-P: 4-strand Kirchmayr–Kessler Prolene^®^ repair.

**Figure 4:**
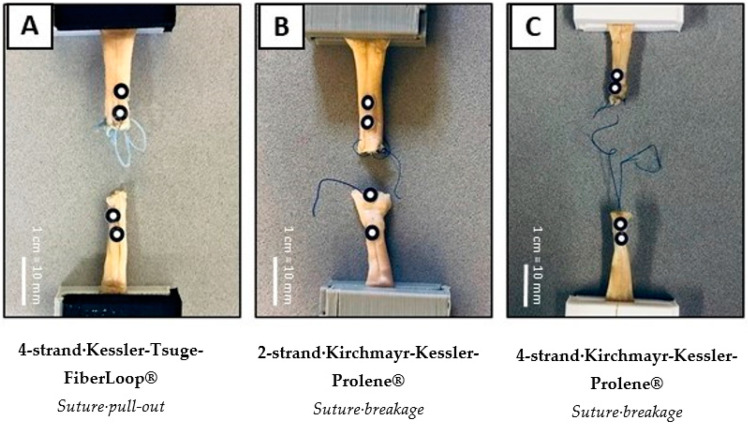
**Examples of the primary failure mode for each repair technique.** (**A**) Suture pull-out being the primary failure mode of 4s-KT-FL; (**B**,**C**) suture breakage being the primary failure mode of 2s-KK-P and 4s-KK-P repairs. 4s-KT-FL: 4-strand Kessler–Tsuge 4-0 FiberLoop^®^ repair; 2s-KK-P: 2-strand Kirchmayr–Kessler Prolene^®^ repair; 4s-KK-P: 4-strand Kirchmayr–Kessler Prolene^®^ repair.

In the cyclic protocol, the 4s-KT-FL repair showed the most variation in failure mode, with suture pull-out at 60% (9/15) being the most dominant; 33% (5/15) of repairs failed by suture breakage right at the origin of the knot and one repair failed due to the slippage of the knot. The primary failure mode of the 2s-KK-P repair was suture breakage at 93% (14/15), and one repair failed by knot slippage at 7% (1/15). The primary failure pattern of the 4s-KK-P repair was also suture breakage at 87% (13/15), and one repair failed by suture pull-out and knot slippage. There was a strong effect between the technique of repair and failure mode (*p* < 0.001; Ф = 0.44). Suture breakage was significantly associated with 2s-KK-P and 4s-KK-P, whereas pull-out was significantly associated with 4s-KT-FL (χ^2^(4) = 34.4, *p* < 0.001). The observed mode of failure distribution is presented in Table 1 and in Figure 3 and Figure 4.

## 4. Discussion

This study provides experimental evidence supporting our hypothesis that the four-strand Kessler–Tsuge repair with FiberLoop^®^ exhibits superior biomechanical properties compared to the two- and four-strand Kirchmayr–Kessler repairs using Prolene^®^. The higher F_max_ and F_2mm_ observed in the 4s-KT-FL repair confirm its suitability for early active mobilization protocols, as hypothesized. As early active mobilization promotes an arrangement of the collagen fibers along the course of primary load application, the formation of adhesions between the tendon and its surrounding sheath is reduced, and intrinsic tendon healing is improved [16,19]. Therefore, early active mobilization plays a crucial role in post-operative tendon recovery and motion. Of note, re-rupture rates approach 4–6% [20], which is why the tendon suture technique and suture material must be chosen with care. Given that, this study provides valuable insights into the suitability of common tendon suture techniques for early active mobilization protocols in the post-operative phase.

### 4.1. Strand Number and the Technique Chosen Have an Effect on the Force at Failure in FDP Repair

According to the literature, early active mobilization exerts forces on the tendon repair, ranging from 30 N to 38 N [17,21]. For the laboratory setting, the suture repair would need to withstand a threshold of 40 N to 50 N of tensile force to tolerate an early active mobilization protocol. The threshold of 40 N to 50 N is based on the literature, indicating that early active mobilization protocols typically exert forces within this range. This ensures that the repair can tolerate physiologically relevant forces during the initial phases of rehabilitation, minimizing the risk of repair failure during post-operative recovery [22,23]. With both the quasi-static and cyclic testing of the given study, the 4s-KK-P repair endured an F_max_ within the range of 50 N until failure, whereas the 4s-KT-FL repair readily surpassed this threshold, indicating sufficient durability. In contrast, the given experiments provided evidence that the 2s-KK-P repair was insufficient in strength, with the lower range of values failing below 40 N. Therefore, the two-strand repair would not be able to tolerate early active mobilization without the risk of failure in the repair within the safety range defined in the literature [17,21].

Previous biomechanical studies showed that the tensile strength of a suture repair is proportional to the number of strands that cross the repair site [24,25]. In comparison with clinical data, a retrospective analysis described a re-rupture rate of 5% for flexor tendons undergoing two-strand suture repairs, while re-rupture rates of four-strand suture repairs were threefold lower at 1.5% [26]. Therefore, current recommendations for FDP repairs are leaning toward core sutures consisting of at least four strands but there are even recommendations toward six strands [27]. However, a higher number of strands also implies more passages of the needle through the tendon, thereby impairing tendon integrity. Furthermore, the suture material proportionally worsens tendon blood supply, impairing tendon healing [28]. In addition, a higher number of strands and excess suture material can be bulky at the repair site, increasing friction at the peritenon [29]. Hence, the ideal repair involves as much suture material as necessary and as little as possible. In the context of the maximum strength, the failure mode also needs to be considered.

In addition to the number of strands, the technique itself influences the repair’s strength. There is evidence that techniques using a looped suture, such as Kessler–Tsuge, distribute tension more evenly across the repair, theoretically reducing localized stress points. However, other findings suggest that the diminished tendon–suture interaction in looped sutures can outweigh this benefit, leading to issues like suture pull-out and reduced tensile strength compared to single-strand sutures with the same number of strands [14,30].

### 4.2. The Kessler–Tsuge Repair Yielded a Higher Force at 2 mm Gap Formation and Differed in Failure Mode Compared to the Kirchmayr–Kessler Repair

Both the 2s- and 4s-KK-P repairs failed primarily by suture breakage, with F_max_ being significantly lower compared to the 4s-KT-FL repair. For quasi-static testing, the primary failure pattern of the 4s-KT-FL repair was suture pull-out at 87%, which is, in fact, a failure of the tendon tissue rather than the suture repair itself. A total of 13% of the 4s-KT-FLrepairs failed due to slippage of the suture knot. Previous studies have already highlighted the issue that FiberWire^®^ knot tends to unravel due to its slightly slippery surface [31]. Therefore, contemporary studies suggest a minimum of six knot throws to prevent knot unraveling [32]. Le et al. [33] showed that five out of ten repairs unraveled with less than six knots. Although a higher number of knots thrown makes repairs with FiberWire^®^ more reliable, an optimal number of knots needs to be found, since each additional knot also adds to the bulkiness of the repair site, thereby impairing tendon gliding. Nevertheless, the 4s-KT-FL repair had a significantly higher maximum force of 63 N from quasi-static testing, with the primary failure attributed to the tendon tissue and not the suture itself.

In addition to F_max_, F_2mm_ is a highly relevant parameter regarding the clinical outcome, as a gap formation of 2 mm or more impairs tendon healing and gliding [34,35,36]. Since an early active mobilization protocol applies forces of 38 N [17], only the 4s-KT-FL repair provided sufficient strength by withstanding 48 N until a 2 mm gap formed under dynamic testing. Both Kirchmayr–Kessler repairs did not tolerate higher loads than 38 N until 2 mm gap formation, and therefore might be unsuitable for early active mobilization in the post-operative phase. Jordan et al. [9] showed that monofilament sutures such as Prolene^®^ lead to gap formation at a significantly lower tension force due to their plastic deformation. FiberLoop^®^, as a braided suture material, had the lowest plastic deformation of all tested suture materials and performed best against suture gapping. Nevertheless, the comparison of F_2mm_ varies slightly between different studies [33], which might be owed to different measurement methods and surgical procedures.

### 4.3. The Cyclic Scenario Well Reflects the Beneficial Findings of the Four-Strand Kessle–-Tsuge Repair

By analyzing the cyclic testing scenario, the number of completed cycles needs to be taken into account as well to mimic the cumulative force that is applied by an early active mobilization protocol over time [17]. The number of completed cycles until failure and the number of cycles at 2 mm gap formation reflect the effect of repetitive stress on the repair site. The 4s-KT-FL repair endured significantly more testing cycles compared to the conventional 2s-KK-P repair, which corresponded to a higher cumulative force. The difference in the number of completed cycles between the 4s-KK-P and 4s-KT-FL repairs did not meet statistical significance.

### 4.4. Limitations

There are some inherent limitations to this study. First, an epitendinous running suture was performed in all groups, which can enhance the ultimate tensile strength up to 20% [37,38]. However, the addition of the running suture was intended to provide findings closely aligned with clinical practice. Second, 2 mm gap formation was only measured at one point, with the assumption of even gapping across the repair. Tahmassebi et al. [39] suggested tracking the gap at multiple points across the repair and averaging the measurements. Using a 3D image correlation system could improve accuracy in future studies. Third, this study was conducted with Thiel-embalmed tendons [40]; F_max_ might be affected by divergent tissue properties compared to unembalmed tendons. While some studies show that Thiel-preserved tendons perform similarly to fresh tendons [41,42], others report differences, particularly in elasticity [43,44,45]. Furthermore, this study did not address other physiological factors involved in tendon healing and adhesion formation that may influence clinical outcomes. Lastly, while the suture techniques were selected to reflect common clinical practice, this study did not directly compare techniques using the same suture material. Future studies could isolate the effects of different suture materials on each technique, particularly in the context of early active mobilization. Investigating how variations in suture materials and configurations affect tendon healing could further refine tendon repair techniques and improve post-operative outcomes.

## 5. Conclusions

The results of this study demonstrate the superior biomechanical properties of the Kessler–Tsuge repair performed with FiberLoop^®^ 4/0 when compared to conventional suture repair with Prolene^®^. This four-strand approach, when paired with the appropriate suture material, appears sufficient to withstand the mechanical stress of early active mobilization protocols. Although a six-strand technique using FiberLoop^®^ would provide an even stronger repair, the increased amount of suture material could impair tendon healing. Moreover, the simplicity of the repair should be also a key consideration, as less surgical trauma to the tendon tissue may promote better recovery. These findings have important implications for surgical practice, as the Kessler–Tsuge repair with FiberLoop^®^ balances between strength and minimized surgical complexity. This approach could lead to better post-operative outcomes and potentially reduce re-rupture rates during early active mobilization. Ultimately, balancing strength and surgical simplicity should guide the selection of the most appropriate technique for clinical application.

## Figures and Tables

**Table 1:** Mode of failure of tendon repair techniques for both testing protocols.

Tendon Repair Technique	Suture Breakage	*p*-Value	Suture Pull-Out	*p*-Value	Slippage of the Knot	*p*-Value
**4s-KT-FL**	16.7% (5/30)	<0.001	73.3% (22/30)	<0.001	10.0% (3/30)	0.1615
**2s-KK-P**	76.7% (23/30)	0.0163	16.7% (5/30)	0.0574	6.7% (2/30)	0.1615
**4s-KK-P**	83.3% (25/30)	<0.001	10.0% (3/30)	<0.001	6.7% (2/30)	0.7641

Association between tendon repairs (4s-KT-FL, 2s-KK-P, 4s-KK-P) and failure mode (suture breakage, suture pull-out, slippage of knot). 4s-KT-FL: 4-strand Kessler–Tsuge 4-0 FiberLoop^®^ repair; 2s-KK-P: 2-strand Kirchmayr–Kessler Prolene^®^ repair; 4s-KK-P: 4-strand Kirchmayr–Kessler Prolene^®^ repair.

## Data Availability

The data supporting the results are available from the authors upon reasonable request.

## Abbreviations

EAM	Early active motion
FDP	Flexor digitorum profundus
2s-KK-P	Two-strand Kirchmayr–Kessler with Prolene
4s-KK-P	Four-strand Kirchmayr–Kessler with Prolene^®^
4s-KT-FL	Four-strand Kirchmayr–Tsuge with FiberLoop^®^
